# Molecular epidemiology of the emerging zoonosis agent Anaplasma phagocytophilum (Foggie, 1949) in dogs and ixodid ticks in Brazil

**DOI:** 10.1186/1756-3305-6-348

**Published:** 2013-12-11

**Authors:** Huarrisson A Santos, Sandra MG Thomé, Cristiane D Baldani, Claudia B Silva, Maristela P Peixoto, Marcus S Pires, Gabriela LV Vitari, Renata L Costa, Tiago M Santos, Isabele C Angelo, Leandro A Santos, João LH Faccini, Carlos L Massard

**Affiliations:** 1 Epidemiology and Public Health Department, IV-UFRRJ, Seropédica, Rio de Janeiro, Brazil; 2 Medicine and Surgery Veterinary Department, IV-UFRRJ, Seropédica, Rio de Janeiro, Brazil; 3 Animal Parasitology Department, IV-UFRRJ, Seropédica, Rio de Janeiro, Brazil; 4 Zootechny Department, ITCA-UFMT, Mato Grosso, Rondonópolis, Brazil; 5 Parasitology Department, ICB-UFMG, Minas Gerais, Belo Horizonte, Brazil; 6 Soils Department, IA-UFRRJ, Seropédica, Rio de Janeiro, Brazil

**Keywords:** *Anaplasma phagocytophilum*, Dogs, Ticks, Epidemiology, Emerging zoonoses

## Abstract

**Background:**

*Anaplasma phagocytophilum* is an emerging pathogen of humans, dogs and other animals, and it is transmitted by ixodid ticks. The objective of the current study was a) detect *A. phagocytophilum* in dogs and ixodid ticks using real-time Polymerase Chain Reaction (qPCR); and b) Determine important variables associated to host, environment and potential tick vectors that are related to the presence of *A. phagocytophilum* in dogs domiciled in Rio de Janeiro, Brazil.

**Methods:**

We tested blood samples from 398 dogs and samples from 235 ticks, including 194 *Rhipicephalus sanguineus* sensu lato, 15 *Amblyomma cajennense*, 8 *Amblyomma ovale* and 18 pools of *Amblyomma* sp. nymphs. A semi-structured questionnaire was applied by interviewing each dog owner. Deoxyribonucleic acid obtained from ticks and dog buffy coat samples were amplified by qPCR (*msp2* gene). The sequencing of *16S rRNA* and *groESL* heat shock operon genes and a phylogenetic analysis was performed. The multiple logistic regression model was created as a function of testing positive dogs for *A. phagocytophilum*.

**Results:**

Among the 398 blood samples from dogs, 6.03% were positive for *A. phagocytophilum. Anaplasma phagocytophilum* was detected in one *A. cajennense* female tick and in five *R. sanguineus* sensu lato ticks (four males and one female). The partial sequences of the *16S rRNA*, and *groESL* genes obtained were highly similar to strains of *A. phagocytophilum* isolated from wild birds from Brazil and human pathogenic strains. The tick species collected in positive dogs were *R. sanguineus* sensu lato and *A. cajennense*, with *A.cajennense* being predominant. Tick infestation history (OR = 2.86, CI = 1.98-14.87), dog size (OR = 2.41, IC: 1.51-12.67), the access to forest areas (OR = 3:51, CI: 1.52-16.32), hygiene conditions of the environment in which the dogs lived (OR = 4.35, CI: 1.86-18.63) and *Amblyomma* sp. infestation (OR = 6.12; CI: 2.11-28.15) were associated with *A. phagocytophilum* infection in dogs.

**Conclusions:**

This is the first report of *A. phagocytophilum* in ixodid ticks from Brazil. The detection of *A. phagocitophylum* in *A. cajennense*, an aggressive feeder on a wide variety of hosts, including humans, is considered a public health concern.

## Background

*Anaplasma phagocytophilum* infects granulocytes, predominantly neutrophils, in which it reproduces, forming colonies termed morulae. This bacterium infects humans, dogs, horses, cats, ruminants, llamas, and a variety of small mammals [1]. The major vectors of *A. phagocytophilum* belong to the *Ixodes persulcatus* complex, including *Ixodes ricinus*, *I. persulcatus*, *Ixodes scapularis* and *Ixodes pacificus*[2,3]. Other ticks have been indicated as vectors, including *Ixodes trianguliceps*, *Ixodes ventalloi*, *Ixodes hexagonus* and *Rhipicephalus turanicus*[2,4-6].

Although *A. phagocytophilum* has been reclassified, investigations based on the molecular bases of the *groEL* and *16S rRNA* genes [7], have shown that *A. phagocytophilum* exhibits some genetic heterogeneity. Genetic variations were found in *16S rRNA*, *groEL*, *msp2*, *msp4* and *Anka* genes in *A. phagocytophilum* isolated from ticks and mammals [8].

Five genetic variants of *A. phagocytophilum* with 1-2 nucleotide differences in the *16S rRNA* sequence were identified in dogs [9]. Organisms detected in dogs from Switzerland had an identical *16S rRNA* gene sequence found in humans with granulocytic anaplasmosis [3], demonstrating that these dogs might serve as hosts of genetic variants of *A. phagocytophilum* able to infect humans.

A number of aspects related to the host, the environment and the agent have been incriminated as risk factors for infection of *A. phagocytophilum* in dogs. These factors include season, co-infections, clinical signs and the genetic variants of the parasite [9]. *Anaplasma phagocytophilum* infections are diagnosed most frequently in months with peaks of nymph and adult stages of the tick vectors [10,11]. Recent studies have shown a greater tendency toward infection in adult dogs, reflecting a higher probability of tick infestation with time [12]. Furthermore, older dogs may be more susceptible.

In Brazil, there are reports of *A. phagocytophilum* infection in dogs from November to May in Rio de Janeiro state [5]. The authors found a strong association between *A. phagocytophilum*-positive dogs and the presence of *Amblyomma* ticks. These data suggest that this tick genus might be involved with *A. phagocytophilum* transmission in this region. Also in Brazil, molecular detection of *A. phagocytophilum* was observed in wild birds and sheep [13,14]. Seroprevalence studies have found evidence of *A. phagocytophilum* infection in small ruminants [15], equines [16] and dogs [17], in Pernambuco state, Notheastern Region of the country, in São Paulo and Paraná state, respectively. Despite this evidence, it is not possible to consider granulocytic anaplasmosis as an endemic disease in Brazil, since studies are isolated and samples are not representative. The aim of this study was to investigate the presence of *A. phagocytophilum* DNA in blood samples and in ixodid ticks collected from dogs using Real-Time PCR and study epidemiological aspects related to the presence of *A. phagocytophilum* DNA in dogs associated with variables related to the host, the environment and the possible tick vectors in the state of Rio de Janeiro, Brazil.

## Methods

### Description of the studied area

This study was carried out in the municipalities of Itaguaí and Seropédica (43° 10’ 376” W and 22° 57’133 “S), respectively, located in the metropolitan mesoregion of the state of Rio de Janeiro, from November 2009 to November 2010.

### Sample size of dogs and ticks

The sampling of dogs in these municipalities was calculated according to a previously described equation [18] with a confidence interval of 95% and an error margin of 3%, assuming an expected prevalence of 10%, based on the data regarding the frequency of *A. phagocytophilum* in Brazil [5]. Three hundred and ninety-eight dogs were selected (Itaguaí, n = 136; Seropédica, n = 262), based on convenience sampling.

To determine the occurrence of *A. phagocytophilum* in a tick population, the sample size was determined considering a prevalence of 10% of infected ticks in an infinite population, based on the value of maximum prevalence reported for *Ehrlichia chaffeensis* in the *Amblyomma americanum* tick [19,20]. Thus, the minimum number of ticks to be tested was 138, assuming an absolute precision of 5% and a confidence interval of 95% were assumed. Nevertheless, 235 ticks were evaluated in this study.

### Epidemiologic questionnaire

A semi-structured questionnaire was applied by interviewing each dog owner to collect information related to the dog and the environment. This information included the following items: sex (female or male); age (<2 years old or ≥ 2 years old); racial definition (defined breed dogs or mixed-breed dogs); dog size as large (50 cm or more) or small/medium (less than 50 cm); history of tick infestation (yes or no); local access of the dog (urban environment/yard/grazing/forest); contact with other animal species (yes or no); acaricide treatment (yes or no); dog habitat as urban or rural (based on the urban perimeter delimited by municipal governments); condition of environment hygiene as unsatisfactory or satisfactory (based on the environment cleanliness and the presence of feces in the location that the dog lives); veterinary assistance (yes or no); tick infestation (presence or absence of ticks at the time of collection of the blood sample); presence of *Amblyomma* spp. (presence or absence of ticks of the genus *Amblyomma* at the time of sample collection); presence of *Rhipicephalus sanguineus* sensu lato (presence or absence of this tick species at the time of sample collection) and presence of ectoparasites (presence or absence of ticks, fleas, lice and mites at the time of collection).

### Buffy coat and tick collection in the dogs

After obtaining the owner’s consent, the animals were restrained for clinical examination and blood collection. A sample of 5 mL of peripheral blood was drawn from each animal by cephalic venipuncture and placed under a vacuum in sterile tubes containing anticoagulant. Buffy coat samples were obtained after centrifugation at 2,500xg for 5 minutes, placed in 1.5 mL micro tubes and stored at-80°C until DNA extraction.

The entire body of each animal was examined for ticks, with particular attention paid to the auricular pavilion region, the head, the neck, the chest, the armpits, the inguinal region and the region under the tail. In infested animals, the level of tick infestation was categorized as follows: low infestation (< five ticks), moderate infestation (≥ five <15 ticks), high infestation (≥15 <30 ticks) and very high infestations (≥30 ticks). The ticks were preserved in tubes containing isopropyl alcohol. The adult tick species were identified using dichotomous keys [21,22]. Nymphal stage ticks were classified to genus level.

Ticks were separated according to the species, sexual dimorphism, developmental stage, animal source, collection date and locality of origin.

### Search for morulae

All dogs sampled were examined for the presence of *A. phagocytophilum* morulae in granulocytes using the blood smear method. Briefly, thin blood smears were prepared; the smears were air-dried and stained using Giemsa and then examined by light microscopy at 1,000 × .

### DNA extraction

The deoxyribonucleic acid (DNA) was extracted from 70 μL of buffy coat using the DNeasy Blood and Tissue kit (Qiagen, Valencia, CA, USA), according to the manufacturer’s recommendations.

Genomic material of adult ticks was extracted from a single specimen, whereas the DNA of nymphs was extracted from a pool of five specimens. DNA was extracted from tick samples according to the protocol described previously [23]. The concentration and purity of the DNA from all blood samples and ticks was determined using spectrophotometry (Nanodrop ND-2000®, Thermo Scientific, Wilmington, DE, USA). DNA samples were diluted to obtain a final concentration of 30 ng/μL.

The positive control of *A. phagocytophilum* was obtained from antigenic substrates in commercial slides prepared for immunofluorescence (Fuller Laboratories, Fullerton, CA, USA). Genomic material in the slides was purified using the DNeasy Blood and Tissue kit (Qiagen, Valencia, CA, USA) according to manufacturer’s recommendations.

### Real-time PCR assay (qPCR)

DNA samples obtained from the dog buffy coat and ticks were analyzed by qPCR with targets in the *msp2* gene of *A. phagocytophilum*[24]. The reactions were performed in triplicate using the Real-Time PCR System StepOnePlus® instrument (Applied Biosystems). The quantification cycle (Cq) was standardized between plates and Cq was manually allocated three cycles after the fluorescent base. Samples with Cq values of less than or equal to 40 cycles were considered as positives. Samples were considered positive for *A. phagocytophilum* if any 1 of 3 replicate samples showed amplified DNA for *A. phagocytophilum* relative to negative controls.

The amplification of *16S rRNA* (546 bp) and *groESL* (1715 bp) heat shock operon genes was performed from positive samples in the qPCR [25,26] for diagnosis confirmation.

### Cloning, sequencing and phylogenetic analysis

The nucleotide sequences of the *16S rRNA* and *groESL* heat shock operon genes were determined for *A. phagocytophilum* samples from 3 ticks and 12 dogs. PCR products were purified, cloned and sequenced [27]. The sequencing was performed on the equipment ABI 3730 DNA Analyzer (Applied Biosystems / Perkin Elmer, CA, USA). The identity of the fragments was analyzed using multiple alignments by the Basic Local Alignment Search Tool (BLAST).

The phylogenetic position of *A. phagocytophilum* isolated from ticks and Brazilian dogs was inferred using the Neighbor-Joining method. The combination of phylogenetic clusters was assessed by bootstrap test with 1000 replicates. The evolutionary distances were calculated by Kimura 2-parameter method. There were a total of 420 nucleotides for the *16S rRNA* gene and 1167 for the *groESL* heat shock operon in the final data set. The analyzes were conducted in MEGA 5.0 [28].

### Analytical sensibility of qPCR to the Anaplasma phagocytophilum diagnostic

The analytical sensitivity of the assay was determined by evaluating serial decimal dilutions of the amplicon cloned into the pGEM-T plasmid [29]. The sensitivity of the real-time PCR assay was evaluated with and without the addition of 1 μL of DNA extracted from ticks (*Amblyomma cajennense*) and a dog buffy coat. The concentration and purity of the plasmid DNA were measured using a spectrophotometer (Nanodrop ND-2000®, Thermo Scientific, Wilmington, DE, USA). The concentration of the plasmid DNA was used to calculate the number of plasmids. A standard curve was constructed with six points representing six serial decimal dilutions ranging from 1 to 100,000 plasmid copies containing a 122-bp fragment of the *msp2* gene of *A. phagocytophilum*. The standard curves were performed with and without the addition of 1 μL of *A. cajennense* DNA (laboratory colony) and the whole blood of an uninfected dog.

### Statistical analysis

The chi-square test or the G test at the 20% level of significance was used to assess whether the presence of *A. phagocytophilum* DNA in the dogs was associated with independent variables collected through the epidemiological questionnaire. The Spearman test was used for those variables exhibiting p <0.20 in the chi-square test or G test to remove the highly correlated variables of the multiple logistic regression analysis. The most biologically important of two highly correlated variables was maintained in the multiple logistic regression analysis.

Independent variables with p <0.20 and ρ <0.7 were included in the multiple logistic regression model as a function of the dogs testing positive for *A. phagocytophilum* using qPCR. An error of 5% was assumed in the final model.

To verify the association level between the positive dogs in qPCR test for *A. phagocytophilum* and the tick infestation level, a frequency ratio was calculated at a 5% significance level using uninfested dogs as a reference variable. This analysis was performed using BioEstat, version 5.0 [30].

A bivariate analysis was performed using BioEstat, version 5.0 [30], and the multiple logistic regression model was conducted using the R statistical software, version 2.11.1 [31].

### Institutional ethical license

These procedures were approved by the Ethics Committee on Research of the Federal Rural University of Rio de Janeiro-UFRRJ (COMEP/UFRRJ), protocol number 124/2011, process number 23083.005908/2011-01.

## Results

Inclusions in neutrophils suggestive of *A. phagocytophilum* were not observed in 398 blood samples analyzed by blood smear. Of all DNA samples tested by qPCR, 24 (6.03%) tested positive for *A. phagocytophilum*. Quantitative PCR was conducted on 194 samples of *R. sanguineus* sensu lato (100 females, 82 males and 12 pools of five nymphs), on 15 samples of *A. cajennense* (nine females and six males), on eight samples of *Amblyomma ovale* (five females and three males) and on 18 pools of *Amblyomma* sp. nymphs. The frequency of *A. phagocytophilum* positive ticks found was 2.55% (n = 6/235). A higher frequency of positive results was observed in *A. cajennense* ticks [one female, 6.67% (n = 1/15)] than in *R. sanguineus* sensu lato ticks [n = 5/194; 7.76% in males (n = 4/194) and 0.51% in females (n = 1/194)]. All positive ticks for *A. phagocytophilum* were collected from negative dogs.

Fifteen positive samples (12 samples obtained from dogs and three from ticks) were cloned and sequenced. All of them showed 100% identity with each other. A single partial sequence of each gene (*msp2*, *16S rRNA* and *groESL*) was submitted to GenBank under the access number [HQ670750, KF836093 and KF836094, respectively].

**Figure 1:**
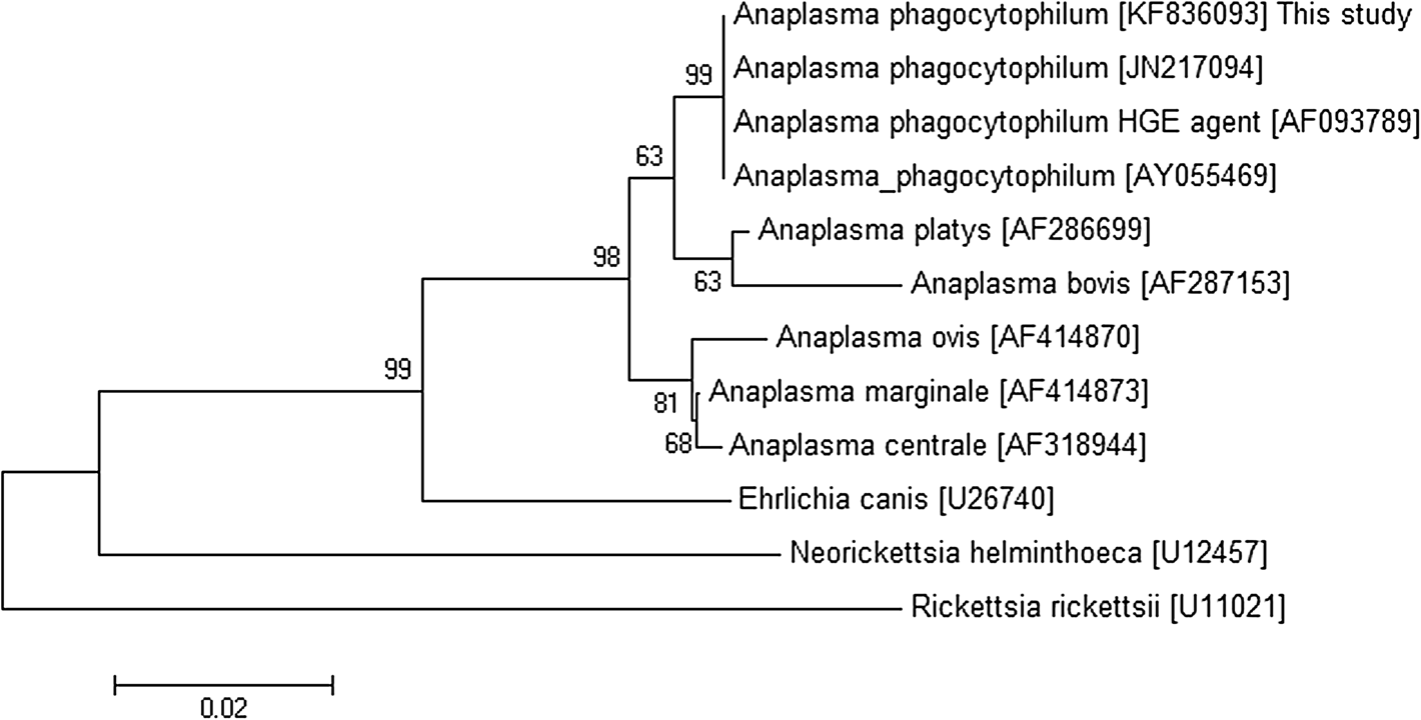
**Phylogenetic dendrogram of *Anaplasma phagocytophilum *isolated from dogs and ticks based on *16S rRNA *gene sequence comparison (420 bp).** GenBank accession numbers are shown in parentheses. The tree was constructed using the neighbor-joining method and numbers above internal nodes indicate the percentages of 1,000 bootstrap replicates that supported the branch.

**Figure 2:**
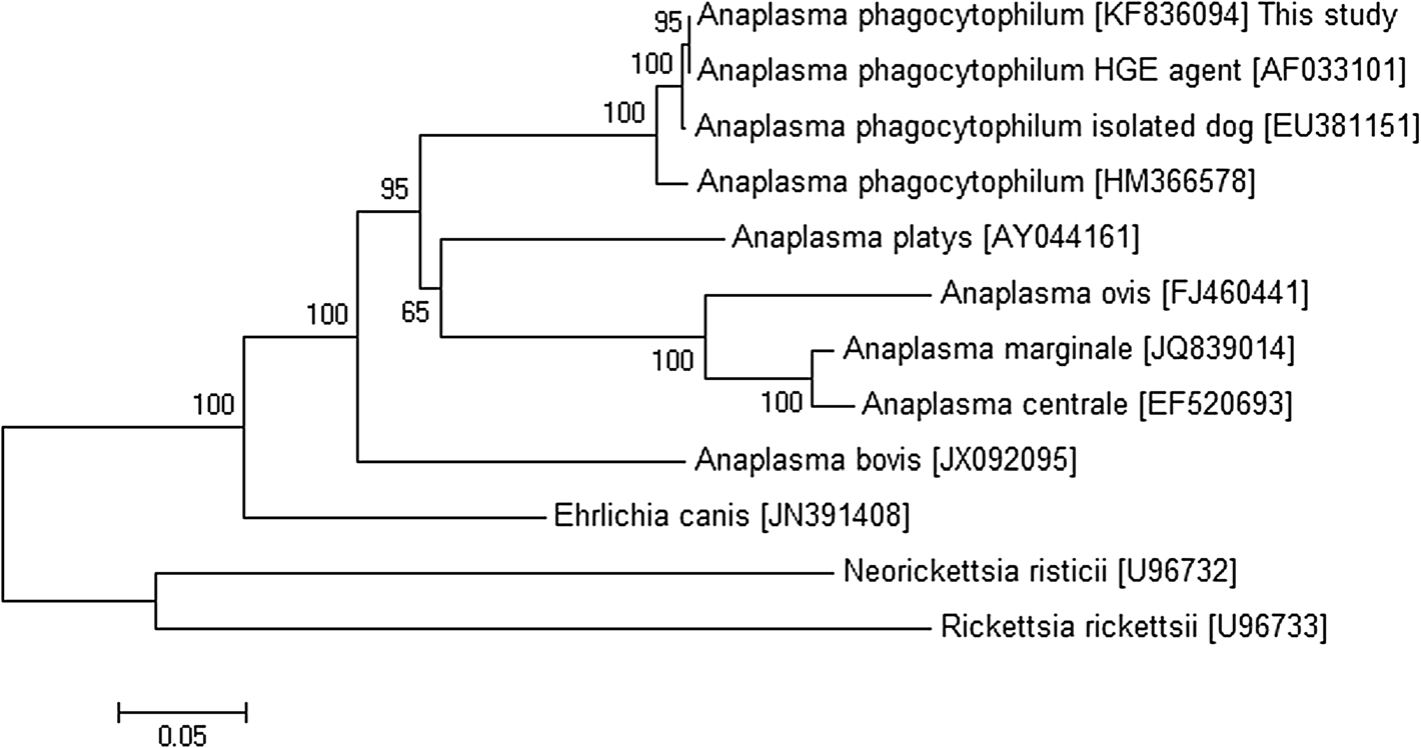
**Phylogenetic dendrogram of *Anaplasma phagocytophilum *isolated from dogs and ticks based on *groESL *heat shock operon gene sequence comparison (1167 bp).** GenBank accession numbers are shown in parentheses. The tree was constructed using the neighbor-joining method and numbers above internal nodes indicate the percents of 1,000 bootstrap replicates that supported the branch.

Of the 24 blood samples positive in qPCR, only 12 were positive for the *16S rRNA* and *groESL* heat shock operon genes. The nucleotide sequences of the *16S rRNA* gene were identical to each other and the same was observed for the *groESL* operon. Regarding the six tick samples, only three were positive for both genes with an identical nucleotide sequence for the *16S rRNA* and *groESL* heat shock operon genes. The *16S rRNA* and *groESL* sequenced genes of *A. phagocytophilum* showed 100% identity with other sequences isolated from dogs (Figures 1 and 2).

**Figure 3:**
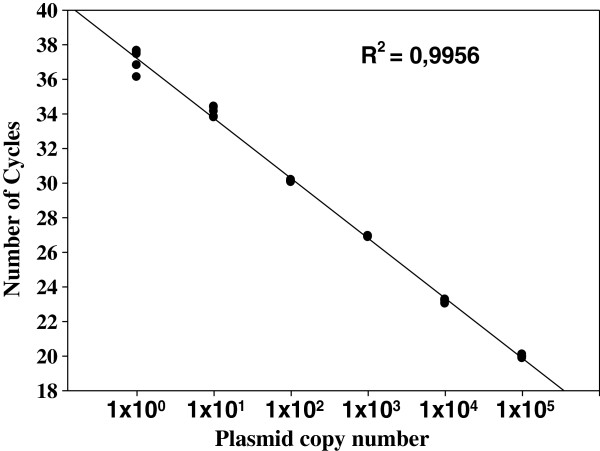
**A standard curve created from serial decimal dilutions of plasmid DNA containing a 122-basepair fragment of the *Anaplasma phagocytophilum msp2 *gene.** The cycle quantification value obtained using real-time PCR was plotted as a function of the initial plasmid copy number.

**Figure 4:**
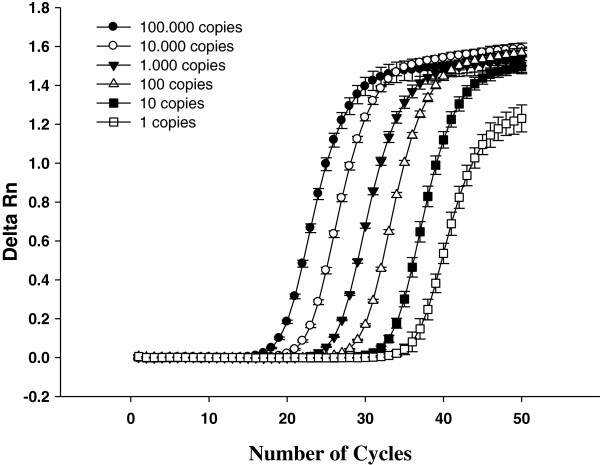
**The analytical sensitivity of the real-time PCR used in the experiment.** The curve shows the amplification of serial dilutions [1 to 100,000 copies] of the plasmid containing the 122-basepair fragment of the *Anaplasma phagocytophilum msp2* gene isolated in dogs and ticks.

The analytical sensitivity of the qPCR technique was evaluated. The detection limit of the technique was one plasmid copy containing the *A. phagocytophilum msp2* gene (Figure 3). No significant difference was found in the analytical sensitivity (p > 0.05) when 1 μL of an equimolar mixture of DNA from buffy coat of an uninfected dog and DNA from an uninfected *A. cajennense* tick were added to the qPCR. High amplification efficiency was demonstrated by identical slopes between amplification curves (Figure 4).

No association was found (p > 0.05) between the variables such as sex, breed, age and animal size, and the positive result in the real-time PCR (Table 1). Tick infestation history was the variable related to the host that was associated with a positive qPCR result (p < 0.05). Dogs with a tick infestation history were 2.86 times more likely to yield positive results in qPCR for *A. phagocytophilum*.

**Table 1:** **The factors associated with *Anaplasma phagocytophilum *** **infection in dogs from municipalities of Itaguaí and Seropédica, Rio de Janeiro State, Brazil, as determined using multiple logistic regression**

**Variables related to the dogs and environment**	**Real-time PCR**		**Bivariate**		**Multivariate**		
	**Dogs sampled**	**Positives (%)**	**χ**^**2**^	**P**	**P**	**OR**	**CI 95%**
**Gender**							
Female	190	4.74	1.073	0.4093	-	-	-
Male	208	7.21					
**Racial definition**							
Mixed Breed	285	5.26	1.042	0.4311	-	-	-
Defined Breed	113	7.96					
**Age**							
< 2 years	130	3.08	3.971	0.1338	-^a^		
≥ 2 years	268	7.46			0.18	-	0.34-6.21
**Dog size**							
Large	33	15.15	5.28	0.0553	0.04	2.43	1.51-12.67
Small/middle	365	5.21			-^a^		
**History of tick infestation**							
Yes	332	7.23	-	0.0488^b^	0.01	2.86	1.98-14.87
No	66	0			-^a^		
**Local access dog**							
Urban environment/yard/grazing	350	4.48	15.585	0.0003	-^a^		
Forest	48	18.75			0.006	3.51	1.52-16.32
**Contact with other species**							
Yes	214	7.94	2.614	0.1599	0.15	-	0.67-10.02
No	184	3.80			-^a^		
**Acaricide treatment**							
Yes	288	5.90	0.030	0.9500	-	-	-
No	110	6.36					
**Dog’s habitat**							
Rural	233	6.44	0.165	0.8476	-	-	-
Urban	165	5.45					
**Condition of environment hygiene where the dog lives**							
Unsatisfactory	223	8.97	6.999	0.0150	0.03	4.35	1.86-18.63
Satisfactory	165	2.42			-^a^		
**Veterinary assistance**							
Yes	167	7.78	1.563	0.2999	-	-	-
No	231	4.76					
**Tick infestation**							
Yes	206	7.77	2.273	0.1946	0.34	-	0.45-24.02
No	192	4.17			-^a^		
**Presence of *Amblyomma *** **sp**							
Yes	82	14.63	13.493	0.0006	0.000	6.12	2.11-28.15
No	316	3.78			-^a^		
**Presence of *Rhipicephalus sanguineus *** **sensu lato**							
Yes	170	5.29	0.284	0.7491	-	-	-
No	228	6.58					

χ2 = Value of Chi-square Test, P = p value, OR = Odds Ratio, CI = Confidence Interval.

aCategory reference.

b*G test*.

A higher percent of infected dogs was observed in males (7.21%, n = 15/208) when compared with females (4.74%, n = 9/190). *Anaplasma phagocytophilum* infection was more frequent (7.46%, n = 20/268) in dogs older than two years. Among positive dogs, 5.26% (n = 15/285) were mongrels, and 7.96% (n = 9/113) were purebreds.

Variables including acaricide treatment, contact with other species, the dog’s habitat [rural or urban] and veterinary care were not associated (p > 0.05) to infection with *A. phagocytophilum* in the dogs. Despite absence of significant association among these variables, the frequency of positive dogs in close contact with other animal species (7.94%, n = 17/214) was higher than dogs that were not exposed to other species (3.80%, n = 7/184). The variable “kind of environment accessed” was associated with *A. phagocytophilum* infection. In this study, 18.75% (n = 9/48) of the dogs that had access to forest areas were infected with *A. phagocytophilum*, whereas 4.48% (n = 15/350) of the dogs that had access to pasture areas/urban environment/backyard were positive when analyzed by qPCR. The hygiene conditions of the environment in which the dogs lived were also associated (OR = 4.35, CI = 1.86-18.63) with *A. phagocytophilum* infection.

The variable “ectoparasite infestation” was removed from the final logistic regression model because of the high correlation (ρ > 0.7) with the variable “tick infestation”. Ectoparasite infestation was observed in 79.16% (n = 19/24) of the positive dogs diagnosed by qPCR. The presence of ticks was verified in 66.67% (n = 16/24) of the positive dogs; the presence of ticks parasiting dogs had no association (p = 0.19) with *A. phagocytophilum* infection. Dogs in which the presence of ticks of the single genus *Amblyomma* was evaluated showed an odds ratio of 6.21 times, demonstrating a strong association with *A. phagocytophilum* infection. The species of ticks found most commonly parasiting positive dogs were *A. cajennense* (50%, n = 12/24) and *R. sanguineus* sensu lato (37.50%, n = 9/24). Co-infestation with *A. cajennense* and *R. sanguineus* sensu lato were observed in 20.83% (n = 5/24) of positive dogs. Among positive dogs for *A. phagocytophilum*, 28.57% (n = 12/42) and 5.29% (n = 9/170) were infested with *A. cajennense* and *R. sanguineus* sensu lato, respectively. No *A. phagocytophilum* DNA amplification was observed in dogs infested with *A. ovale*, *A. dubitatum* or nymphs of *Amblyomma* sp.

The frequency ratio between the infestation levels by ticks and *A. phagocytophilum* infection in dogs ranged from 1.7 to 3.0 with increasing infestation level (Table 2). The level of infestation was evaluated without considering the tick species found on the dogs.

**Table 2:** **The frequency ratio of dogs positive for *Anaplasma phagocytophilum *** **based on real-time PCR analysis according to the tick infestation level in dogs from the municipalities of Itaguaí and Seropédica, Rio de Janeiro State, Brazil**

**Infestation level**	**Real-time PCR**		**Frequency ratio**		
	**Dogs sampled (n)**	**Positive (%)**	**FR**	**P**	**IC**
Uninfested	192	4.17	-^a^	-	-
Low infestation	112	4.46	1.07	0.43	0.36-3.20
Moderate infestation	58	5.17	1.24	0.48	0.34-4.53
High infestation	28	10.71	2.57	0.15	0.72-9.12
Very high infestation	8	12.50	3.00	0.40	0.42-21.19

^a^Reference category, FR-Frequency ratio, P = p value, IC-Confidence Interval.

## Discussion

The negative result for *A. phagocytophilum* in the analysis of blood smears can be explained by the short period of bacteremia, less than 28 days [32]. In experimental infections, the morulae of *A. phagocytophilum* could be seen in neutrophil cytoplasm from 4 to 14 days post-infection and could be observed for a period of 4 to 8 days [33]. Infected neutrophil percent in the acute phase of the disease varied from 1 to 42% [9,11,33], and probably affected blood smear diagnosis, since in the present study, none of the dogs showed clinical symptoms. During blood smears analyses, one aspect that needs attention is the *A. phagocytophilum* misdiagnosis based only on intracytoplasmic inclusions in neutrophils, since *Ehrlichia ewingii* species, present in dogs from Brazil [34], have similar morphological characteristics when compared with *A. phagocytophilum*[35]. Accordingly, PCR analysis is the best choice for a diagnostic test, and it is the most reliable and specific evaluation for granulocytic ehrlichiosis diagnosis [36].

The *16S rRNA* gene fragment [KF836093] amplified from DNA of blood from dogs and ticks samples showed 100% identity with sequences isolated from wild birds [JN217096, JN217094 and JN217095] in Brazil [13]. These results showed that *A. phagocytophilum* circulates in Brazil and has wild birds and dogs as hosts. *16S rRNA* gene sequences isolated from Brazilian dogs and ticks showed 100% identity with the prototype strain isolated from humans [U02521] in northern Minnesota and Wisconsin [37] and from dogs in Germany [JX173652] [38]. Five variants of *A. phagocytophillum* have been found in dogs; however, co-infection with more than one variant can be observed in some cases, including the strain what occurs in humans.

The partial sequences of the *groESL* [KF836094] heat shock operon isolated from dogs and ticks were similar to each other but not identical to *A. phagocytophilum* strains isolated from humans and dogs in Florida [CP006617 and CP006618] due to one transition from A to T [37]. A sequencing artifact cannot be excluded in this case, but it seems to be unlikely, as the PCR was performed with high fidelity DNA polymerase and sequencing was performed three times in both directions from cloned PCR products.

The current study analyzed characteristics of dog blood samples infected with *A. phagocytophilum*. The results demonstrated no statistical association with age, although a significant number of positive dogs (83.33%, n = 20/24) belonged to the group with two years or more. In Sweden, the percentage of seropositive dogs increased with age [39], reflecting an increased likelihood of exposure to infected tick vectors over time. The average age of dogs infected with *A. phagocytophilum* reported in the literature is approximately six to eight years old [9,40-42].

At the final logistic regression model, the variable dog size was associated with the presence of *A. phagocytophilum* DNA in dogs (OR = 2.41, IC: 1.51-12.67). Most likely, the size of the dog did not influence the susceptibility of infection with *A. phagocytophilum*, but rather the habits or purpose of the breed; frequently, the dogs in this study were kept in the vicinity of the home as guard animals or as hunting dogs. Hunting exposes dogs to forest areas and several tick species, which increases the risk of infection by vector-borne pathogens, including *A. phagocytophilum*.

*Anaplasma phagocytophilum* is retained in the natural cycle between rodents and ticks of the genus *Ixodes*[43,44]. In the studied area, the species *Ixodes amarali* has been detected parasitizing opossums of the genus *Didelphis*[45]; there are no reports of dog parasitism by the *Ixodes* species in the state of Rio de Janeiro, despite the fact that several surveys have been conducted during the last decade. It was found that *A. phagocytophilum* infection in dogs was associated with the tick infestation history reported by the owners; an identical finding was observed in Taipei, Taiwan [46]. This association was not observed in relation to the presence of ticks on dogs at the time of collection, although a tendency for *A. phagocytophilum* infection was verified in dogs infested with ticks at the time of collection (66.67%). In the multiple logistic regression model, tick species that belonged to the genus *Amblyomma* were strongly associated (OR = 6.12; CI: 2.11-28.15) with *A. phagocytophilum* infection in dogs. This association did not occur for dogs infested with *R. sanguineus* sensu lato (p = 0.75). A number of studies have shown different results regarding tick exposure history as reported by owners. For example, tick infestation has not been described in any of the dogs examined for granulocytic anaplasmosis in western Washington, USA [9].

In a study in Sweden, exposure to ticks was observed in 13 of 14 dogs examined [40]. In Germany, a similar result was described, and tick infestation was observed in 80% of 18 dogs naturally infected with *A. phagocytophilum*[42].

In the state of Rio de Janeiro, the species of *Amblyomma* that most frequently infest dogs are: *A. cajennense*, *Amblyomma aureolatum* and *A. ovale* in that order [47,48]. These tick species are most often found parasitizing dogs in rural areas near secondary forests, a variable that was associated (OR = 3:51, CI: 1.52-16.32) with the presence of *A. phagocytophilum* DNA in dogs. The current study showed that *A. cajennense*, *Amblyomma dubitatum* and *A. aureolatum* were found only in dogs that had access to forest areas. Tick species parasitizing dogs in Brazil are diverse because there are many different ecosystems in the country. The environmental characteristics and the diversity of host species present in each area are key factors for the variety and abundance of the species of ticks that infest dogs.

This is the first report of *A. phagocytophilum* infection in *R. sanguineus* sensu lato and *A. cajennense* adult ticks in Brazil. In this study, despite the epidemiological evidence suggesting the genus *Amblyomma* as a possible vector of *A. phagocytophilum*, *R. sanguineus* sensu lato exhibited a higher absolute number of positive ticks. This result is most likely associated with the small number of ticks from the genus *Amblyomma* analyzed by qPCR. *Anaplasma phagocytophilum* has been detected by PCR in *R. turanicus* in Italy with a frequency of 1.8% [6], a higher rate was observed in the present study (2.58%). The only report of *A. phagocytophilum* infection in ticks from the genus *Amblyomma* was described in Poland with *Amblyomma flavomaculatum*, parasiting lizards imported from Africa [49].

The rate of infection in *R. sanguineus* sensu lato male ticks was higher than that observed in female ticks; this result was also reported in a study in Jilin province, China and Tunisia, Africa [19,50]. Nevertheless, in a study conducted in Poland [51], the number of female ticks positive for *A. phagocytophilum* (45.7%, n = 79/173) was higher than that of male ticks (4.5%, n = 9/202) and nymphs (0.9%, n = 3/319). Further studies using a larger number of ticks at different seasons and in different locations and habitats are necessary to understand the distribution and variation in the rate of *A. phagocytophilum* infection in ticks.

## Conclusions

In the evaluated region, the epidemiology of *A. phagocytophilum* infection includes large sized dogs; animals which have access to forest areas; dogs in contact with other animals; animals that live in poor hygienic conditions; and, particularly, dogs infested with *Amblyomma* sp. ticks.

## Abbreviations

CGA: Canine granulocytic anaplasmosis; CI: Confidence interval; Cq: Quantification cycle; DNA: Deoxyribonucleic acid; FAM: 6-carboxy-fluorescein; OR: Odds ratio; qPCR: Real time polymerase chain reaction; TAMRA: 6-carboxy-tetramethylrhodamine.

## Competing interests

The authors declare that they have no competing interest.

## Authors’ contributions

HAS: Developed the conception and design of the study, carried out the laboratory work, data analysis, intellectual interpretation and writing of the manuscript. SMGT, LAS and CDB: supervised the study, carried out the laboratory work, intellectual interpretation and critical revision of the manuscript for publication. CBS, MPP, MSP, GLVV, RLC, TMS, ICA: collected blood samples from dogs, samples of ticks, applied the epidemiological questionnaire and drafted the manuscript. JLHF and CLM: Supervised the study, and were involved in intellectual interpretation and critical revision of the manuscript for publication. All authors read and approved the final manuscript.
